# Estimation of Time Since Death From Morphological Changes Occurring in White Blood Cells After Death

**DOI:** 10.7759/cureus.89773

**Published:** 2025-08-11

**Authors:** Anurupa Choudhury, Anindita Mahanta

**Affiliations:** 1 Forensic Medicine, Dhubri Medical College, Dhubri, IND; 2 Physiology, All India Institute of Medical Sciences, Guwahati, Guwahati, IND

**Keywords:** autolytic changes, leukocytes, non-refrigerated cadavers, post-mortem changes, time of death

## Abstract

There are several methods to estimate time since death, but no method, used singly, is totally reliable. After death, several morphological changes occur in all the cellular components of blood, including the white blood cells. In this study, blood samples were collected from 20 non-refrigerated cadavers, with known time and cause of death and without any hematological, oncological, or infectious disease, brought to the mortuary of a tertiary care hospital. One blood film was prepared and stained immediately, and subsequent blood films were made and stained at regular intervals up to 120 hours. It was observed that lymphocytes were most resistant and neutrophils were most vulnerable to autolytic changes following death. Observing the sequential changes occurring in the leucocytes in the postmortem period may serve as an additional parameter in determining the time since death.

## Introduction

The interval between death and the time of examination of a body is known as postmortem interval (PMI). Determination of the PMI is of critical importance in the practice of forensic medicine as it is important for both civil and criminal cases [1]. The exact time of death cannot be fixed by any one method, but only an approximate range can be given, because there are considerable variations in individual cases [1,2]. The longer the postmortem period, the wider the range of the estimate, i.e., the estimate of the PMI becomes less accurate. In determining the time of death, all available history should be taken and then local physical or environmental factors at the scene of crime should be noted [2]. The changes that occur in the body after death include cooling of the body, cadaveric lividity or postmortem hypostasis, rigor mortis, progress of decomposition, adipocere and mummification, entomology of the cadaver, quantity and digestive state of the gastric contents, contents of urinary bladder, biochemical changes in electrolyte and enzyme levels in various body fluids (such as blood, cerebrospinal fluid, synovial fluid, and vitreous humor), and changes in muscle enzyme levels [1,2]. Morphological changes in the white blood cells could provide additional help in estimating the time since death. However, not much emphasis has been laid on the study of cellular changes occurring in the postmortem period. Following somatic death, due to the lack of oxygen, accumulation of carbon dioxide, and change in hydrogen ion concentration, irreversible changes occur in the internal environment of the body, which affects all the cellular elements of blood, including the white blood cells [3]. Changes in the morphology of the white blood cells can be easily studied with the help of a peripheral blood smear, and the sequence of changes that occur in the cells over time can serve as an additional tool to determine the time since death. A few studies have been conducted to study the pattern and sequence of changes occurring in white blood cells in the postmortem period. As early as 1981, Laiho and Penttila [4,5] reported their findings regarding autolytic changes in blood cells. Using the vital dye exclusion test, they demonstrated that the loss of viability of white blood cells showed a moderate correlation with the increase in the postmortem period. Furthermore, on examination of the morphology of various types of cells in the peripheral blood of human cadavers, they found rapid deterioration of the staining properties and marked morphological changes in many leucocytes. Following this, further studies were conducted by Babapulle and Jayasundera [6], Dokgoz et al. [7], Bardale and Dixit [8], and Kumar et al. [3], to name a few. With this background, the present study was conducted to evaluate the sequential morphological changes occurring in the WBCs in the postmortem period as an adjuvant method for estimation of PMI.

## Materials and methods

The study was conducted at a tertiary care hospital of Assam in North-East India. The study protocol was reviewed and approved by the Institutional Ethics Committee. The study sample consisted of 20 cases of medicolegal autopsies conducted in the Department of Forensic Medicine and Toxicology. Because of official and administrative procedural delay, most of the cases are received for postmortem examination after 12 hours of death, and limited cases are received within 12 hours of death. This accounted for the small study sample size.

The study included cases of medicolegal autopsies in which the time of death was known, cause of death could be ascertained after autopsy, the first sample of blood could be collected within 24 hours of death, and age of the deceased was between 20 and 60 years. Known cases of septicemia, hematological disorders, malignancy, infectious diseases and charred bodies were excluded from the study.

For all cases included in the study, 2 mL of peripheral blood was collected in an ethylenediaminetetraacetic acid (EDTA) vial at the time of autopsy. From this collected blood, a blood smear was made immediately and stained with Leishman’s stain. It was then labelled according to the known PMI. The blood samples were stored at room temperature to simulate prevailing environmental conditions. Subsamples were taken from this from this blood sample at pre-determined intervals up to 120 hours (at 9, 12, 18, 24, 36, 48, 72, 96, 120 hours) in order to prepare blood smears to observe the sequential changes occurring in the leucocytes after death. These slides were labeled by adding the period of in vitro storage to the known PMI. All the blood smears were stained with Leishman’s stain and observed under the oil immersion objective. A total of 100 leucocytes were counted, and the appearance of the following changes were noted: nuclear pyknosis, nuclear fragmentation, cytoplasmic vacuolation, and cellular breakdown. Observations were categorized into nine groups of PMIs, ranging from 0-6 hours, 6-12 hours, 12-18 hours, 18-24 hours, 24-36 hours, 36-48 hours, 48-72 hours, 72-96 hours, and 96-120 hours.

## Results

Out of the 20 cases included in the study, seven cases had PMI between 0 and 6 hours, eight cases between 6 and 12 hours, three between 12 and 18 hours, and two between 18 and 24 hours. Table 1 lists the cases included in the study along with the PMI and the cellular changes observed in the granulocytes. Among the granulocytes, morphological changes started earlier in the eosinophils compared to the neutrophils. The first change observed was nuclear pyknosis, which appeared between 0 and 6 hours, followed by nuclear fragmentation, which appeared between 6 and 12 hours, and, finally, cytoplasmic vacuolation, which appeared after 12 hours. Among the agranulocytes, monocytes showed nuclear pyknosis between 12 and 18 hours and nuclear fragmentation between 18 and 21 hours. Lymphocytes were found to be normal up to 21 hours.

**Table 1:** PMI and morphological changes in granulocytes PMI, postmortem interval

Case no.	PMI (in hours)	Neutrophils	Eosinophils
1	3	Normal morphology	Normal morphology
2	4	Normal morphology	Pyknosis (<50%)
3	4	Normal morphology	Pyknosis (<50%)
4	5	Pyknosis (<50%)	Pyknosis (>50%)
5	5	Pyknosis (<50%)	Pyknosis (>50%)
6	6	Pyknosis (<50%)	Pyknosis (>50%)
7	6	Pyknosis (<50%)	Pyknosis (100%), nuclear fragmentation (<50%)
8	7	Pyknosis (>50%)	Pyknosis (100%), nuclear fragmentation (<50%)
9	8	Pyknosis (>50%)	Pyknosis (100%), nuclear fragmentation (<50%)
10	8	Pyknosis (>50%)	Pyknosis (100%), nuclear fragmentation (<50%)
11	9	Pyknosis (100%)	Pyknosis (100%), nuclear fragmentation (<50%)
12	10	Pyknosis (100%), nuclear fragmentation (<50%)	Pyknosis (100%), nuclear fragmentation (<50%)
13	10	Pyknosis (100%), nuclear fragmentation (<50%)	Pyknosis (100%), nuclear fragmentation (<50%)
14	11	Pyknosis (100%), nuclear fragmentation (<50%)	Pyknosis (100%), nuclear fragmentation (>50%)
15	12	Pyknosis (100%), nuclear fragmentation (<50%)	Pyknosis (100%), nuclear fragmentation (>50%), cytoplasmic vacuolation (<50%)
16	14	Pyknosis (100%), nuclear fragmentation (>50%)	Pyknosis (100%), nuclear fragmentation (>50%), cytoplasmic vacuolation (<50%)
17	14	Pyknosis (100%), nuclear fragmentation (>50%)	Pyknosis (100%), nuclear fragmentation (>50%), cytoplasmic vacuolation (<50%)
18	18	Pyknosis and nuclear fragmentation (100%), cytoplasmic vacuolation (<50%)	Pyknosis and nuclear fragmentation (100%), cytoplasmic vacuolation (>50%)
19	20	Pyknosis and nuclear fragmentation (100%), cytoplasmic vacuolation(>50%)	Pyknosis and nuclear fragmentation (100%), cytoplasmic vacuolation (>50%)
20	21	Pyknosis and nuclear fragmentation, cytoplasmic vacuolation (100%)	Pyknosis and nuclear fragmentation, cytoplasmic vacuolation (100%)

Follow-up slides were prepared from subsamples of stored blood (Table 2). The slides were further examined under oil immersion after staining with Leishman’s stain.

**Table 2:** Preparation of sub-samples of stored blood PMI, postmortem interval

PMI (hours)	Time after death when follow-up slides were made (PMI + in vitro storage period) (hours)
0-6	9, 12, 18, 24, 36, 48, 72, 96, 120
6-9	12, 18, 24, 36, 48, 72, 96, 120
9-12	18, 24, 36, 48, 72, 96, 120
12-18	24, 36, 48, 72, 96, 120
18-24	36, 48, 72, 96, 120

Up to 24 hours after death, the morphological changes observed in the white blood cells in the slides made from in vitro stored blood were similar to the changes occurring in vivo following death.

At a PMI between 24 and 36 hours, neutrophils appeared grossly dysmorphic with nuclear pyknosis and fragmentation and cytoplasmic vacuolation, the cell membrane was intact, eosinophils were grossly dysmorphic with cell membrane breakdown occurring in <50% of observed cells but were still identifiable, monocytes showed nuclear pyknosis and fragmentation in >50% of the observed cells but cytoplasmic vacuolation was seen in <50% of the observed cells, and lymphocytes showed nuclear pyknosis in <50% of the observed cells. At a PMI between 36 and 48 hours, the neutrophils showed disintegration of the cell membrane but were still identifiable, the eosinophils were not identifiable on the smears examined, the monocytes were grossly dysmorphic with nuclear pyknosis, fragmentation, and cytoplasmic vacuolation in >50% of observed cells, the cell membrane was intact, and lymphocytes showed nuclear pyknosis in >50% of the observed cells with nuclear fragmentation in <50% cells. At a PMI between 48 and 72 hours, the neutrophils were no longer identifiable on the smears examined; the eosinophils were no longer identifiable, the monocytes started showing breakdown of the cell membrane but were still identifiable, and lymphocytes showed gross nuclear abnormalities in the form of pyknosis and fragmentation in >50% of cells observed. At a PMI between 72 and 96 hours, the neutrophils and eosinophils were no longer identifiable, the monocytes were no longer identifiable, the lymphocytes were grossly dysmorphic with nuclear pyknosis, fragmentation, and cytoplasmic vacuolation seen in >50% of the observed cells, and the cell membrane was intact. At a PMI between 96 and 120 hours, the neutrophils, eosinophils, and monocytes were no longer identifiable, and lymphocytes showed breakdown of the cell membrane but were still identifiable. Beyond 120 hours, lymphocytes could no longer be identified on smears. From the above findings, a rough estimate of the time since death can be made (Table 3).

**Table 3:** Estimate of the time since death from changes in cellular morphology of leukocytes PMI, postmortem interval

Changes observed in cellular morphology	PMI (in hours)
Pyknosis of neutrophils and eosinophils	0-6
Pyknosis and nuclear fragmentation of neutrophils and eosinophils	6-12
Pyknosis and nuclear fragmentation in neutrophils. Pyknosis, nuclear fragmentation, and cytoplasmic vacuolation in eosinophils. Pyknosis in monocytes.	12-18
Pyknosis, nuclear fragmentation, and cytoplasmic vacuolation in neutrophils and eosinophils. Pyknosis and nuclear fragmentation in monocytes.	18-24
Grossly dysmorphic neutrophils with intact cell membrane. Grossly dysmorphic eosinophils with breakdown of the cell membrane. Pyknosis, nuclear fragmentation, and cytoplasmic vacuolation in monocytes. Lymphocytes show pyknosis.	24-36
Grossly dysmorphic neutrophils with breakdown of the cell membrane. Eosinophils not seen. Grossly dysmorphic monocytes with intact cell membrane. Pyknosis and nuclear fragmentation in lymphocytes (<50%).	36-48
Neutrophils and eosinophils not seen. Grossly dysmorphic monocytes with breakdown of the cell membrane. Pyknosis and nuclear fragmentation in lymphocytes (>50%).	48-72
Neutrophils, eosinophils, and monocytes not seen. Pyknosis, nuclear fragmentation, and cytoplasmic vacuolation in lymphocytes with intact cell membrane.	72-96
Neutrophils, eosinophils, and monocytes not seen. Grossly dysmorphic lymphocytes with breakdown of the cell membrane.	96-120
No identifiable leukocytes.	>120

## Discussion

In the present study, the earliest change was observed in the eosinophils in the form of nuclear pyknosis at 4 hours PMI, followed by the appearance of nuclear fragmentation at 6 hours PMI. Cytoplasmic vacuolation was first seen at 12 hours PMI. Beyond 24 hours, eosinophils were grossly dysmorphic with breakdown of the cell membrane but identifiable. Eosinophils were no longer identifiable beyond 36 hours. Penttila and Laiho [5] reported seeing eosinophils in 71% cases within a postmortem time of upto 24 hours but in only 10-20% cases beyond 120 hours. Babapulle and Jayasundera [6] reported finding identifiable degenerated eosinophils upto 36-60 hours PMI but none beyond 60-66 hours. Bardale and Dixit [8] and Kumar et al. [3] reported identifiable changes in the morphology of eosinophils between 0-6 hours PMI and inability to identify eosinophils on smears beyond 24 hours PMI. Dokgoz et al. [7] observed identifiable changes in eosinophils at 6 hours PMI, and eosinophils could no longer be identified on smears beyond 72 hours PMI.

Postmortem changes in the morphology of neutrophils were first observed at 5 hours PMI in the present study. This is comparable to the findings of Bardale and Dixit [8], Kumar et al. [3], and Dokgoz et al. [7]. However, Bardale and Dixit [8] and Kumar et al. [3] reported that neutrophils could not be identified on smears beyond 24 hours and 36 hours, respectively, while in the present study, neutrophils could be identified on smears up to 48 hours. Dokgoz et al. [7] reported identifiable neutrophils on smears up to 96 hours. Penttila and Laiho [5] reported finding identifiable neutrophils in 80% cases upto 120-168 PMI and even beyond, which is much more than that reported in the present study. Babapulle and Jayasundera [6] reported finding identifiable degenerate neutrophils upto 66 hours PMI but not beyond 66 hours, which is closer to the values reported in the present study.

The present study found the monocytes to be more resistant to the effect of postmortem changes, with appearance of abnormalities between 12 and 18 hours PMI and being identifiable in smears up to 72 hours. Bardale and Dixit [8] and Kumar et al. [3] reported changes in monocytes as early as within 6 hours PMI and absence of identifiable monocytes on smears beyond 18-20 hours PMI. Penttila and Laiho [5] reported finding identifiable monocytes upto 168 hours PMI and even beyond, whereas Babapulle and Jayasundera [6] reported identifiable degenerate monocytes upto 66 hours only and not beyond.

The lymphocytes were found to be most resistant to the effect of postmortem changes. In the present study, changes were first observed at 24 hours PMI, with grossly dysmorphic but identifiable lymphocytes on smears seen at 96 hours. However, lymphocytes could no longer be identified at 120 hours PMI. This is similar to the findings of Dokgoz et al. [7]. Bardale and Dixit [8] and Kumar et al. [3] reported early appearance of morphological changes in lymphocytes at around 10-12 hours PMI, with failure to identify lymphocytes on smears beyond 30-36 hours. Penttila and Laiho [5] reported finding several compact, well-preserved, and quite normally staining small lymphocytes upto 270 hours PMI. Babapulle and Jayasundera [6] reported that finding only identifiable degenerate lymphocytes on the peripheral smear indicated a PMI between 66 to 84 hours. The present study found that the presence of only lymphocytes (dysmorphic but identifiable) on the peripheral smear correlated with a PMI of 72 to 120 hours.

The difference in the appearance of the morphological changes between the present study and that of Bardale and Dixit [8] and Kumar et al. [3] could be due to the difference in the methodology of the study. In the present study, the longest PMI in which blood was taken directly from the cadaver was 21 hours, beyond which time interval blood stored in EDTA vials was used to prepare smears and observe the morphological changes. However, even upto 21 hours PMI, no observable morphological changes were seen in the present study. The methodology of the present study is similar to that of Dokgoz et al. [7], which may account for the concurrence of the results. However, the overall pattern and sequence of changes observed is similar in all the studies, with neutrophils showing the earliest changes and lymphocytes being the most resistant to postmortem autolytic changes.

The sequence of morphological changes occurring in the white blood cells in the postmortem period can, thus, provide a reasonable estimate of the time since death and serve as an additional test in estimating the time since death. Although a reasonable window of time can be determined during which the various cellular changes occur in the different WBCs, there can be instances in which there is early cell lysis or preservation of cellular morphology for a longer period of time. In such cases, the terminal events preceding death and the antecedent cause of death need to be considered.

Strengths of the study

To the best of our knowledge, this is the first study conducted in North-East India. The study explores an easy and inexpensive adjuvant method for the determination of PMI and provides insights into a less explored area of forensic medicine.

Study limitations

The major limitation of the present study is the small sample size owing to the limited number of cases that satisfied the inclusion and exclusion criteria. Also, due to small sample size, it is difficult to comment on the effect of factors such as environmental temperature and humidity on the appearance and progression of changes in the white blood cells. A larger sample size with an even distribution of cases throughout the year is required to consolidate the findings of the present study.

## Conclusions

Following somatic death, irreversible changes occur in the internal environment of the body due to the lack of oxygen, build-up of carbon dioxide, and other toxic metabolites, which lead to cellular death. These changes, in which the cells lose their normal morphology as a result of postmortem autolysis and putrefaction, affect all the cellular elements of blood, including the white blood cells. The sequence of changes from normal morphology to the stage of non-identification can serve as a useful tool for estimating the PMI. Although there are many methods to estimate the time of death, none of the methods, used alone, can provide a reliable estimate of the time of death. The best strategy is to combine the available methods to correlate the findings to estimate the time since death. In this context, use of the morphological changes occurring in the leucocytes in the postmortem period to estimate the time since death may serve as an additional tool.
